# Pseudotumor Cerebri Postpartum: A Case Report

**DOI:** 10.7759/cureus.32893

**Published:** 2022-12-24

**Authors:** Ali Msheik, Rami Atat, Safaa Al Arab

**Affiliations:** 1 Neurological Surgery, Al Zahraa Hospital University Medical Center (UMC), Choueifat, LBN; 2 Medical Sciences, Lebanese University, Hadath, LBN; 3 Neurology, Al Zahraa Hospital University Medical Center (UMC), Beirut, LBN

**Keywords:** elevated intracranial pressure, nausea and vomiting in pregnancy, postpartum headache, acetazolamide, pseudotumor cerebri syndrome (ptcs)

## Abstract

Idiopathic intracranial hypertension (IIH), called pseudotumor cerebri, could cause postpartum headaches. Generally, this diagnosis is idiopathic and treatment is mainly medical to avoid serious complications of possible vision loss. In this paper, we report the case of a 24-year-old lady who developed a similar constellation of symptoms and was diagnosed with this condition. Postpartum, the patient demonstrated symptoms of headache and vision disturbances. Workup ruled out infectious processes and intracranial pathologies. Normal cranial magnetic resonance imaging (MRI) and high cerebrospinal fluid (CSF) pressure during lumbar puncture led to a diagnosis of IIH. Initiation of medication allowed rapid improvement of symptoms and evaded imminent morbidity. Further discussion in light of the latest findings of the literature is held after the presentation of the case. This case sheds light on the importance on importance of fundoscopy in patients demonstrating new-onset headaches especially postpartum with the absence of intracranial pathologies.

## Introduction

Idiopathic intracranial hypertension (IIH), also called pseudotumor cerebri, could cause the postpartum headache and is commonly identified after ruling out other common causes of headaches [1,2]. Although rare during pregnancy, pseudotumor cerebri is well documented in women of childbearing age more than in other populations [2]. Studies suggest venous outflow pathologies as the etiology of IIH, yet this disease remains mostly idiopathic in nature and can be according to some reports a cause of increased venous pressure in the brain [3]. Vasogenic extracellular brain edema and a low conductance of cerebrospinal fluid (CSF) outflow at the arachnoid villi may be the cause of the development of increased CSF pressure in IIH [4].

High BMI increases the risk for IIH, particularly in women. Obesity elevates intraabdominal pressure, which elevates pleural pressure and cardiac filling pressures, obstructing venous return from the brain and resulting in increased intracerebral venous pressure and intracranial pressure [5].

Intracranial hypertension IIH or primary pseudotumor cerebri syndrome (PTCS) is identified when brain parenchyma is normal in the absence of ventriculomegaly, mass lesion, venous sinus thrombosis, underlying infection, or neoplasms [6]. The two factors that exacerbate existing IIH are pregnancy and exogenous estrogens. There are no harmful impacts on the fetus that have been noted in pregnant women having Intracranial hypertension. There is no rise in the number of fetal losses at early diagnosis, so trying to decrease disease progression by therapeutic abortion is not stated [7]. The probability of recurrence of IIH does not raise in subsequent pregnancies [8].

This syndrome has various symptoms: The most common symptom is headache, and other associated symptoms present are transient visual obscurations, pulsatile tinnitus, and papilledema with loss of vision which is a serious complication in untreated conditions [9,10]. 

The Modified Dandy criteria for IIH used in Idiopathic Intracranial Hypertension Treatment Trials (IIHTT) are signs and symptoms of increased intracranial pressure, absence of localizing findings on neurologic examination, and absence of deformity, displacement, or obstruction of the ventricular system and normal neuro-diagnostic studies, except for increased CSF pressure (>200 mm water) [11]. Abnormal neuroimaging except for empty sella turcica, optic nerve sheath with filled-out CSF spaces, and smooth-walled non-flow-related venous sinus stenosis or collapse should lead to another diagnosis [11].

Obstetric indications dedicate the delivery modality where either has not been correlated to different outcomes relative to IIH. However, concurrent papilledema along with vigorous uterine contractions can promote the former and cause permanent eye damage and possibly vision loss [12,13]. To evade the aforementioned complication, medications are initiated including steroids, and diuretics, namely acetazolamide. More to add, lumbar punctures in IIH patients are beneficial because it reduces CSF pressure by allowing CSF drainage [4,5]. These therapies during pregnancy are safe with no risk of teratogenicity [10,12-14].

## Case presentation

We present the case of a 25-year-old female patient received at the emergency department for severe headache and fever that started two days before presentation. The patient has a history of a Cesarean section (C-section) procedure done one month prior to the presentation. The C-section procedure was electively done due to the preference of the patient. otherwise, the patient denied any other surgical or medical history. The pregnancy was not complicated and all prenatal screening and laboratory workup was done with no significant findings.

Upon arrival at the emergency department, the patient was conscious, cooperative, and oriented. She had a fever of 38.6 degrees, non-focal headache, mild nausea, and decreased oral (per os) intake two days before the presentation. The patient's skin was mildly dry. Otherwise, no rash was evident.

The patient reported neck pain. Otherwise, the past medical history was negative for any pathologies, diseases, and surgical interventions besides the C-section procedure. The patient denied a history of allergies to food or medications. Physical examination revealed mild nuchal rigidity, bilateral papilledema, mild photophobia, equally reactive pupils, and negative Brudzinski and Kernig signs. The significant results of the laboratory workup are depicted in Table 1.

**Table 1:** Significant laboratory workup and results of each parameter CSF: cerebrospinal fluid; mg: milligrams; g: grams; dL: deciliter; L: liter; µL: microliter

Laboratory parameter	Normal range	Result
Hemoglobin	12-16 g/dL	11.10
Platelets	150-400 10^3^/µL	100
White blood corpuscles	4-11 10^3^/µL	7.32
Neutrophils	40-68%	68.72
C-reactive protein	0-5 mg/L	9.07
Creatinine	0.67-1.17 mg/dL	0.59
CSF-Glucose	32-82 mg/dL	62.10
CSF-protein	0.1-0.5 g/L	0.269

**Figure 1:**
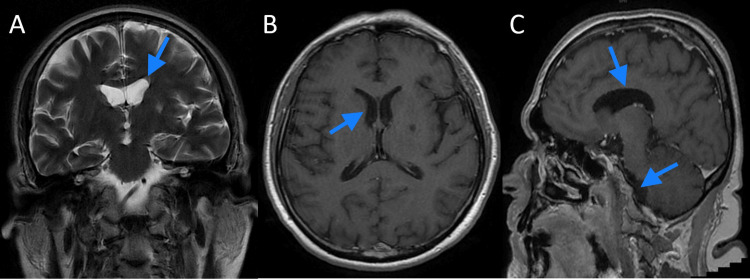
Cranial magnetic resonance imaging of the patient with contrast A: Coronal cut T2 sequence; B: axial cut T1 sequence with contrast; (C) sagittal cut T1` sequence with contrast. Blue arrows show ventricles without evidence of hydrocephalus. Courtesy of ZHUMC radiology department, Beirut, Lebanon.

Blood, urine, and CSF workup and cultures revealed no evidence of ongoing bacterial infection. It was evident that the patient had elevated CSF opening pressure during a lumbar puncture. Polymerase chain reaction (PCR) for COVID-19 virus, herpes simplex virus 1 (HSV-1), HSV-2, varicella-zoster virus (VZV), cytomegalovirus (CMV), Epstein-Barr virus (EBV), human herpesvirus 6 (HHV-6), HHV-7, HHV-8, and the human enteroviruses (HEVs) were all negative. Of note, the aforementioned viruses are usually recommended for testing for encephalitis and are termed neuro9 PCR [15]. Cranial magnetic resonance imaging was done with gadolinium intravenous injection and revealed no ongoing pathology as in Figures 1A-1C. 

Empirically after the withdrawal of blood and due to the initial high index of suspicion of ongoing central nervous system infection, namely meningitis or encephalitis, the patient was started on methylprednisolone, vancomycin, ceftriaxone, and acyclovir. vancomycin was then switched after a few hours onto teicoplanin. However, given the negative laboratory and radiological workup and in the light of the elevated CSF opening pressure on the lumbar puncture and the bilateral papilledema, the patient was diagnosed with IIH postpartum, i.e., postpartum pseudotumor cerebri.

All antiviral and antibiotics were discontinued. Steroids were discontinued. the patient was started on acetazolamide 250 mg per os twice daily after which in 24 hours the patient reported regression of her photophobia and headache. Three days later, the papilledema subsided, and the patient was discharged home on acetazolamide 250 mg twice daily for two weeks. on follow-up, the patient reported discontinuing the medication due to “feeling cured.”

## Discussion

After the exclusion of various etiologies of headache postpartum, IIH namely pseudotumor cerebri can be the reason for headaches two to three weeks after delivery. Although termed idiopathic, the theory of venous perfusion abnormalities and drainage is credible in the explanation of the pathophysiology behind pseudotumor cerebri [1]. The claim of CSF drainage abnormalities through the arachnoid villi has been suggested as an explanation of this pathology along with venous sinuses stenosis [16]. In light of the limited evidence of sinuses stenosis in pseudotumor cerebri, stenting has been limited to a small population of patients [16].

Risk factors for pseudotumor cerebri include obesity and female gender [17]. IIH is reported to worsen due to elevated levels of estrogen both exogenous or endogenous due to pregnancy IIH [16]. Our patient was asymptomatic during pregnancy. Although the literature reports an increased incidence of recurrence among pregnancies of a female with IIH, no reports of affection for the fetus are found [17].

Because 10% of IIH patients present with headaches, and papilledema that can result in permanent vision loss, treatment is mainly symptomatic and preventive [18]. Acetazolamide is the mainstream treatment [17]. As it allows a decrease of the volume of CSF by promoting diuresis and due to the limited side effect spectrum being limited to oral or limb paresthesias, and weight gain beyond the dose of 500 mg per day [18], acetazolamide was ideal for our patient and improved her headache after 24 hours of initiation. Papilledema was improved three days after treatment with acetazolamide. The duration of treatment is not standardized and in most cases is guided by the symptomatology of the patients [18,19]. In this case, the patient stopped the medication after three weeks on her own. Examination during follow-up revealed normal eye examination and no report of headache or any neurological deficit.

In the literature, postpartum IIH was documented. Mathew et al. reported a case of postpartum IIH [1]. However, the delivery process was laborious and complicated with postpartum pneumonia. Similarly, the patient had headaches and visual disturbances which were resolved upon initiation of acetazolamide medication.

## Conclusions

In conclusion, a prompt fundoscopic examination is crucial for every patient with new-onset headaches. IIH must be one of the primary differential diagnoses for every woman with new-onset headache postpartum due to the high risk of blindness associated with the disease. Our patient presented postpartum with headaches and visual disturbances. Delays related to radiological and laboratory workup could have delayed diagnosis and caused permanent visual damage. Fundoscopy for patients with new onset headaches with no clear organic causes should be a routine step and here comes the essence of publishing this case.
